# Sexual dimorphism in hormone–gut microbiome associations across reproductive states in wild giant pandas

**DOI:** 10.1002/imo2.70108

**Published:** 2026-06-05

**Authors:** Qinlong Dai, Xinyuan Cui, Chenyi Gao, Xiaoxiao Tang, Kaizhi Wen, Minhui Shi, Chen Zhang, Yue Liu, Xinru Yang, Qiuyu Huang, Lifeng Zhu

**Affiliations:** ^1^ Shimian Management and Protection Station, Giant Panda National Park Sichuan China; ^2^ School of Medicine Nanjing University of Chinese Medicine Nanjing China; ^3^ Liziping Giant Panda's Ecology and Conservation Observation and Research Station of Sichuan Province Nanchong China

## Abstract

Gut microbiota–host endocrine interactions in wild mammals remain poorly understood. Giant pandas experience seasonal dietary shifts and constrained annual reproduction, offering an ideal model to investigate microbiome–endocrine coupling. We integrated fecal hormone profiling, microsatellite‐based individual identification, and gut microbiome sequencing in wild giant pandas. Microbial diversity and composition varied seasonally, with α diversity peaking during bamboo shoot‐feeding. Hormonal dynamics followed predictable patterns: elevated estrogen during estrus, sustained progesterone post‐estrus, and higher cortisol during leaf/stem‐feeding periods. Hormone levels significantly correlated with microbial taxa and diversity, indicating coordinated microbiome–endocrine dynamics. Notably, microbiome–hormone coupling was significant in males but not in females, and males exhibited greater hormonal and microbial variability. Our findings demonstrate seasonal restructuring of gut microbiome and endocrine profiles in wild giant pandas with sex‐dependent coupling, providing an integrated framework for non‐invasive conservation monitoring.

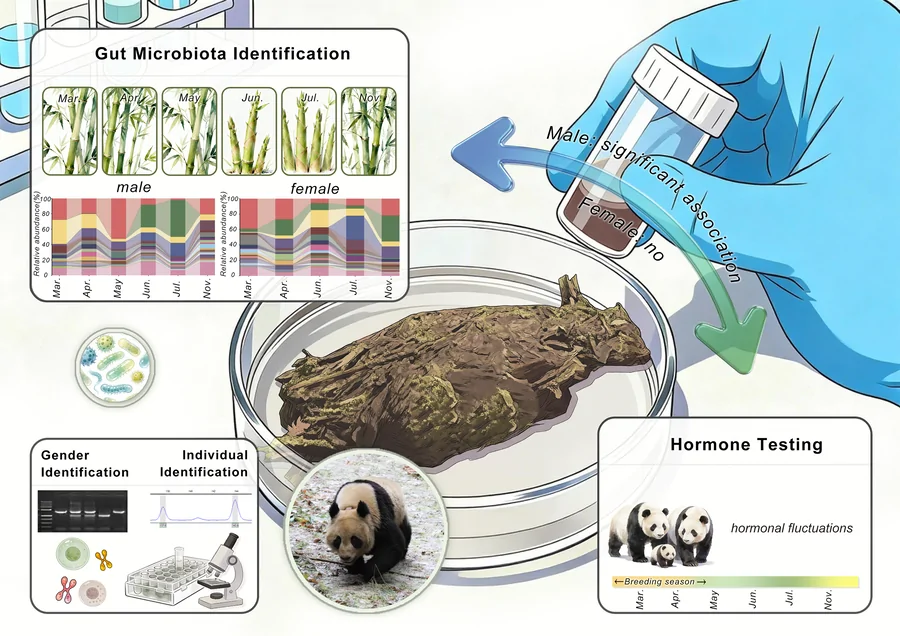

## AUTHOR CONTRIBUTIONS

**Qinlong Dai**: Conceptualization; methodology; writing—original draft. **Xinyuan Cui**: Methodology; software; writing—original draft. **Chenyi Gao**: Software; formal analysis; writing—original draft. **Xiaoxiao Tang**: Data curation; formal analysis. **Kaizhi Wen**: Software; formal analysis. **Minhui Shi**: Formal analysis. **Chen Zhang**: Formal analysis. **Yue Liu**: Data curation. **Xinru Yang**: Formal analysis. **Qiuyu Huang**: Data curation. **Lifeng Zhu**: Conceptualization; methodology; funding acquisition; writing—original draft; writing—review & editing.

## CONFLICT OF INTEREST STATEMENT

The authors declare no conflicts of interest.

## ETHICS STATEMENT

This study involved noninvasive fecal sample collection from giant pandas and complied with all relevant ethical guidelines for wildlife research. The study protocol was reviewed and approved by the Laboratory Animal Ethics Committee of Nanjing University of Chinese Medicine (Approval No. ACU240113). All procedures were conducted in accordance with the guidelines of the institution to minimize disturbance to the animals and their habitats.

To the editor,

The gut microbiome is a key aspect of host physiology, ecological adaptation, and health [1, 2], but its temporalization with endocrine processes is not fully studied in wild mammals. Such a disparity is specifically pertinent to giant pandas (*Ailuropoda melanoleuca*), having strong seasonal morphine eating patterns and having a strictly limited yearly reproductive cycle [3]. Despite the prior researches that have independently recorded the seasonal changes in gut microbiota or endocrine status in giant panda, the ongoing assessment of microbiome–hormone interaction throughout seasons and across different sexes has been inadequate [4, 5, 6]. In this context, with a combination of fecal hormone, sex, individual genotype, and gut microbiome profiling, we have characterized seasonal microbiome and endocrine variations in wild giant pandas and have tested whether they are coupled.

## 1 INDIVIDUAL IDENTIFICATION AND DATASET OVERVIEW

A total of 104 wild giant panda fecal samples were collected. After DNA extraction, microsatellite amplification, and genotype quality filtering, 62 samples were retained for individual identification and assigned to 32 individuals, including 16 males, 12 females, and 4 individuals of undetermined sex (Table S1). Three identified individuals matched previously released individuals in the Liziping National Nature Reserve. This individual‐level framework enabled us to analyze microbiome–hormone relationships at both sample and individual scales, while accounting for sex‐specific patterns in ecological and physiological variation. These data provide a rare non‐invasive, longitudinal perspective on endocrine and gut microbial dynamics in a wild endangered mammal.

## 2 SEASONAL RESTRUCTURING OF THE GUT MICROBIOME

The gut microbiome of wild giant pandas showed monthly dynamics in both diversity and composition. Alpha diversity was relatively higher in June and July, corresponding to the bamboo shoot‐feeding period (Figure S1A). In March, April, and November, sex‐based alpha diversity differed but was not statistically significant (Figure S1B–D). Beta diversity analysis further supported significant difference of microbial communities across months (Figure S2A,B), with detectable but not statistically significant sex‐associated separation in March and November (Figure S2C,D).

At the genus level, dominant taxa exhibited clear seasonal succession. *Pseudomonas* was more abundant in March and April, *Clostridium sensu stricto 1* dominated in May, while *Acinetobacter* and *Lactococcus* increased during June and July (Figure S3A). Male and female pandas exhibited distinct community features and also showed taxonomic differences across months (Figure S3B,C). The analysis of differences revealed overall variations in the gut microbiota across months (Figure S3D). Linear discriminant analysis Effect Size (LEfSe) further revealed compositional differences between males and females (Figure S3E–G).

These patterns are consistent with the established effect of bamboo‐part preference on giant panda gut microbiota [7, 8]. Bamboo shoots are richer in protein, whereas leaves and stems contain more cellulose, lignin, and flavonoids [9]. Increased alpha diversity during the shoot‐feeding period agrees with previous reports linking this dietary phase to greater microbial diversity [7]. The enrichment of *Pseudomonas* during leaf/stem‐feeding periods may relate to cyanide detoxification and lignocellulose‐associated metabolism in bamboo‐consuming pandas [10, 11], whereas higher abundances of *Acinetobacter* and *Lactococcus* in June–July may reflect a shift toward taxa associated with nutrient assimilation under a protein‐rich seasonal diet [12, 13].

## 3 SEASONAL HORMONE DYNAMICS IN FEMALE AND MALE PANDAS

Hormone levels also varied markedly across months in both sexes (Figure S4). In females, estrogen peaked in March, consistent with the known estrous window of giant pandas, and progesterone remained relatively high from March to June (Figure S4A,B). Cortisol was elevated in March, April, and November (Figure S4C), and testosterone also showed seasonal changes (Figure S4D). Estrogen and progesterone could be detected in males and varied among months (Figure S4E,F). In males, Cortisol was significantly elevated during the bamboo leaf/stem‐feeding period compared with the shoot‐feeding period (Figure S4G), while testosterone was higher in March and again in July (Figure S4H).

These findings are consistent with the past literature regarding endocrinology that female giant pandas exhibit a short‐term annual period of estrus with peak levels of estrogen, which is followed by a significant level of progesterone during the post‐ovulatory phase. The reproductive activation in the breeding season is also supported by male testosterone being higher in March [5]. This high cortisol level in the leaf/stem–feeding months could be evidence of the higher physiological load in the months of nutritional inferiority, but other ecological stressors of the season can also be involved [6, 14]. Collectively, these endocrine patterns indicate that wild giant pandas undergo integrated reproductive and metabolic adaptations throughout the nutritional cycle throughout the year.

## 4 ASSOCIATIONS BETWEEN HORMONE LEVELS AND GUT MICROBIAL FEATURES

To evaluate microbiome–endocrine coupling, we analyzed correlations between hormone and gut microbial features. Network and Spearman analyses revealed extensive positive and negative associations between microbial taxa and hormones (Figure 1A,B and Figure S5). Testosterone showed significant negative correlations with *Escherichia*‐*Shigella* and *Comamonas*, whereas cortisol was negatively correlated with *Yersinia*, *Stenotrophomonas*, *Pedobacter*, and *Turicibacter*. In contrast, progesterone was positively associated with *Leifsonia*, *Massilia*, and *Paeniglutamicibacter*, and estrogen was positively associated with *Leifsonia* but negatively associated with *Turicibacter* and *Clostridium sensu stricto 1* (Figure 1B). Microbial alpha diversity was positively correlated with progesterone and estrogen but negatively correlated with cortisol and testosterone, with significant relationships observed for progesterone and cortisol (Figure 1C). Notably, *Leifsonia*, which was associated with both estrogen and progesterone, was more abundant during March–April, corresponding to the estrous period (Figure 1D).

**Figure 1:**
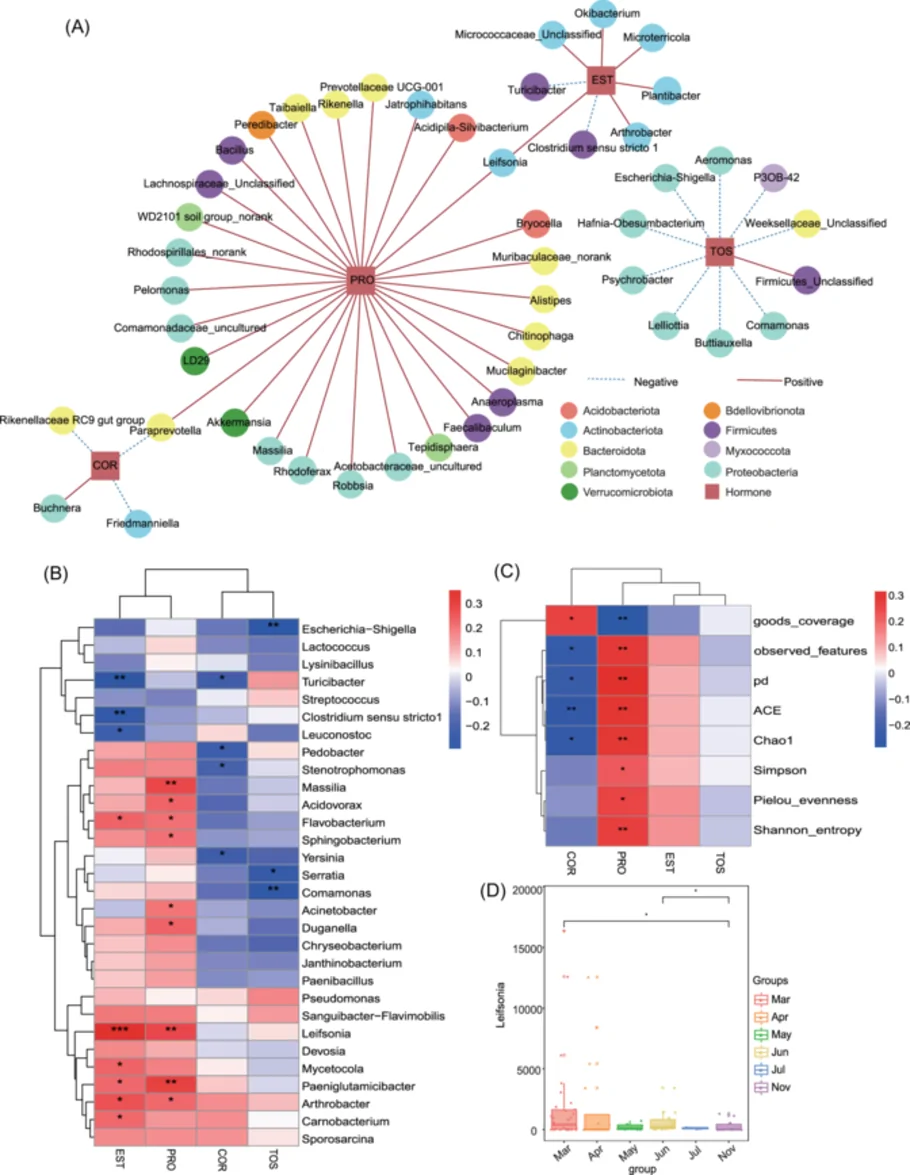
Association between gut microbiome and hormone levels in giant pandas. COR, cortisol; EST, estrogen; PRO, progesterone; TOS, testosterone. *p < 0.05, **p < 0. 01, ***p < 0.001. (A) Correlation network visualized by Cytoscape, illustrating interactions between gut microbial taxa and hormone levels. (B) Heatmap of Spearman correlation coefficients between gut microbial genera and hormones. (C) Heatmap of Spearman correlation coefficients between gut microbial alpha diversity with hormone levels. (D) Relative abundance of Leifsonia across months.

These findings are biologically plausible given growing evidence that gut microbes can interact with steroid metabolism and host endocrine regulation [15, 16]. Microbial enzymes such as β‐glucuronidases may influence estrogen reactivation, while steroid‐transforming bacteria may alter the host hormonal milieu [16, 17]. Conversely, endocrine signals can shape the gut microbiome through immunological and metabolic pathways, including Hypothalamic–Pituitary–Adrenal‐axis‐mediated stress responses [18]. Although our results do not demonstrate causality, they support the existence of a seasonally dynamic microbiome–endocrine association in wild giant pandas.

## 5 SIGNIFICANT MICROBIOME–ENDOCRINE COUPLING IN MALES

We next assessed microbiome–hormone coupling at both sample and individual scales. Euclidean distance‐based hormone profiles were significantly correlated with Bray–Curtis‐based gut microbiome composition at the whole‐sample level (Figure 2A), and this relationship remained significant in males (Figure 2B), but not in females (Figure 2C). Correspondingly, males exhibited greater variability than females in both hormone profiles and gut microbial composition at the sample level (Figure 2D,E). Similarly, at the individual level, significant microbiome–hormone coupling was detected for all identified individuals and for males specifically (Figure 2F,G), whereas the association in females was not significant (Figure 2H). Males exhibited greater variability than females in both hormone profiles and gut microbial composition at the individual levels (Figure 2I,J).

**Figure 2:**
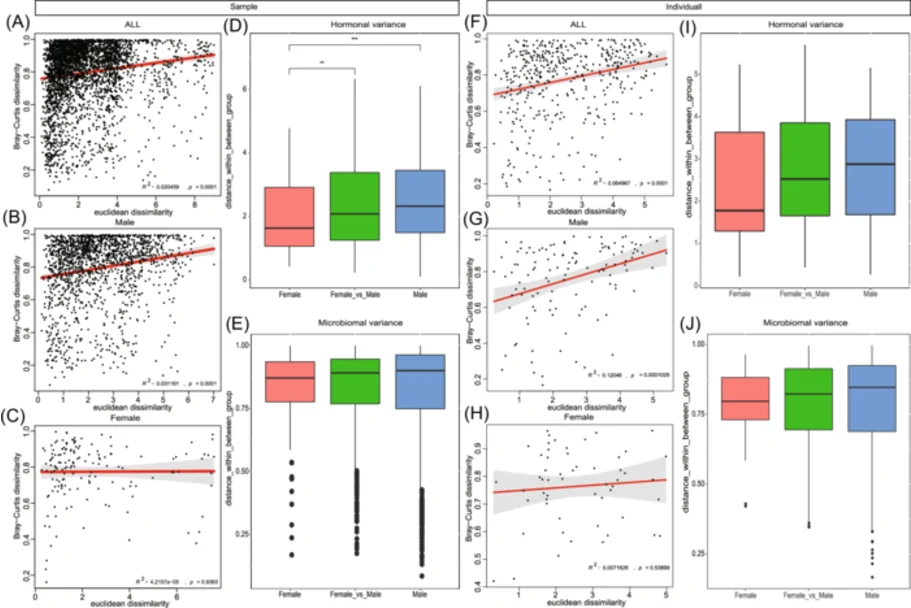
Associations between gut microbiome composition and hormone profiles across different sex samples and individuals. **p < 0. 01, ***p < 0.001. Correlation analysis between hormone‑based Euclidean distance and microbiome‐based Bray–Curtis distance for (A) all samples, (B) male samples, and (C) female samples. (D) Hormone profile dissimilarity based on Euclidean distance in female and male intergroup samples. (E) Gut microbiome composition dissimilarity based on Bray–Curtis distance in female and male intergroup samples. Correlation analysis between hormone‐based Euclidean distance and microbiome‐based Bray–Curtis distance for (F) all individuals, (G) male individuals, and (H) female individuals. (I) Hormone profile dissimilarity based on Euclidean distance in female and male intergroup individuals. (J) Gut microbiome composition dissimilarity based on Bray–Curtis distance in female and male intergroup individuals.

Male giant pandas generally occupy larger home ranges and show stronger territorial and roaming behavior than females, which may amplify inter‐individual differences in diet, environmental exposure, and stress experience. These factors could jointly modulate both endocrine activity and gut microbial composition, leading to greater overall variation in males. However, the sample size for females was relatively small, which may cause the observed differences to be less pronounced.

## 6 IMPLICATIONS FOR CONSERVATION PHYSIOLOGY

To conclude, wild giant pandas exhibited seasonal and gender changes in gut microbiome composition and endocrine state, as well as sex‐specific microbiome–hormone interaction, which was significant in males. Our results build upon prior research on the microbiota of the giant panda gut and subsequent endocrinology, and help combine the two systems in the context of seasonal changes. They endorse the notion that gut microbial ecology could be linked to endocrine adaptations to wild mammals that have profound nutritional and reproductive demands annually. Such an integrated microbiome endocrine viewpoint can assist in optimizing additional non‐invasive surveillance of reproductive state, stress condition, and health management of giant panda conservation.

## Supporting information


**Figure S1:** Variation in gut microbiome alpha diversity across months and between sexes in giant pandas.
**Figure S2:** Variation in gut microbiome beta diversity across months and between sexes in giant pandas.
**Figure S3:** Variation in gut microbiome composition across months and between sexes in giant pandas.
**Figure S4:** Hormone level variations in female and male giant pandas across different physiological stages.
**Figure S5:** Correlation network visualized by Cytoscape, illustrating interactions between gut microbial taxa and hormone levels in male giant pandas.

**Table S1:** The sample information.

## Data Availability

The raw data of the sequences have been submitted to NCBI: PRJNA1393895 (https://www.ncbi.nlm.nih.gov/bioproject/PRJNA1393895). Codes are available at GitHub: https://github.com/ChenyiGao-orange/gut-microbiome. Supplementary materials (methods, figures, tables, graphical abstract, slides, videos, Chinese translated version, and updated materials) may be found in the online DOI or iMeta Science http://www.imeta.science/imetaomics/.
