# Supplementary material for: Sexual dimorphism in hormone–gut microbiome associations across reproductive states in wild giant pandas
Source: IMetaOmics. 2026 Jun 5;3(3):e70108. doi: 10.1002/imo2.70108 (PMC13674390; doi:10.1002/imo2.70108)
Supplement: Supplementary file 1 [file IMO2-3-e70108-s002.docx]

Supporting Information to

**Sexual dimorphism in hormone–gut microbiome associations across reproductive states in wild giant pandas**

**Running title: Hormone–microbe dimorphism in giant pandas**

Qinlong Dai^1, 2, 3, #^, Xinyuan Cui^2, #^, Chenyi Gao^2, #^, Xiaoxiao Tang^2^, Kaizhi Wen^2^, Minhui Shi^2^, Chen Zhang^2^, Yue Liu^2^, Xinru Yang^2^, Qiuyu Huang^2^, and Lifeng Zhu^2, 3*^

^1^ Shimian Management and Protection Station, Giant Panda National Park, Sichuan 625400, China

^2^ School of Medicine, Nanjing University of Chinese Medicine, Nanjing 210023, China

^3^ Liziping Giant Panda’s Ecology and Conservation Observation and Research Station of Sichuan Province, Nanchong 637000, China

^#^ These authors contributed equally: Qinlong Dai, Xinyuan Cui, Chenyi Gao

^*^Correspondence: zhulf2020@126.com (Lifeng Zhu)

***MATERIALS AND METHODS***

**Sample Collection**

104 fresh fecal samples were collected from the Xiaoxiangling Mountains between March and July and in November 2024. Fecal freshness was assessed based on surface mucus characteristics and environmental conditions to estimate defecation time, with fresh samples (≤ 3 days old) prioritized for collection. Feces with intact structure and little difference in gloss between the light–exposed and ground–facing sides were considered approximately 1 day. If the feces are intact, the gloss on the light–exposed side had disappeared or was not obvious, but the surface closest to the ground was glossy, the freshness was judged to be 2–3 days. Conversely, feces lacking luster on the light–exposed side and showing cobweb–like strands or fungal hyphae on the ground–facing side were estimated to be 4–15 days old. Among these, only one female specimen was obtained in both June and July. Samples of undetermined sex were excluded from sex–associated analyses. Each sample was immediately placed in a sterile disposable bag and labeled with geographical coordinates (latitude, longitude), elevation, collection time, and species. The collected samples were frozen and subsequently shipped on dry ice to the laboratory for further processing. Detailed sample data are provided in Table S1.

**DNA extraction and 16S rRNA sequencing**

Collected fecal samples were soaked in anhydrous ethanol for 12 h, followed by centrifugation to rich the pellet for subsequent DNA extraction. Genomic DNA was then extracted using the QIAamp DNA Stool Mini Kit (Qiagen, Germany), in strict accordance with the manufacturer’s protocol. Primers targeting the full–length 16S rRNA gene (27F–1492R) were used for amplification, with each primer pair containing unique barcode sequences. PCR was carried out using the KOD One PCR Master Mix (TOYOBO Life Science). Amplified products were purified with VAHTSTM DNA Clean Beads (Vazyme, Nanjing, China) and quantified with the Qubit® dsDNA HS Assay Kit on a Qubit 3.0 Fluorometer (Invitrogen, Thermo Fisher Scientific, USA). SMRTbell libraries were prepared from the amplified DNA by SMRTbell Express Template Prep Kit 2.0 according to the manufacturer’s instructions (Pacific Biosciences). Purified SMRTbell libraries from the pooled and barcoded samples were sequenced on a PacBio Revio platform using binding kit 2.0.

**Gender determination**

PCR amplification was performed using giant panda zinc finger protein (ZF) gene primers (GTf, 5’–CAAGGATTTCCATGTAGCAGT–3’; GTr, 5’–ACTCTCCACAATGTCTACGGT–3’) [1] under the following conditions: initial denaturation at 95°C for 5 min, 35 cycles of denaturation at 94°C for 50 s, annealing at 59°C for 45 s, and extension at 72°C for 1 min, followed by a final extension at 72°C for 10 min. The amplified products were resolved on a 2% agarose gel at 100 V for 25 min. Specimens showing only the 354bp band (X chromosome–specific) were identified as female, while those exhibiting both the 135 bp (Y chromosome–specific) and 354 bp bands were classified as male.

**Microsatellite identification**

Using 12 microsatellite primer sets, amplification was performed in multiplex groups of three primers each: Set 1: Ame–μ10, Ame–μ13, Ame–μ26; Set 2: Ame–μ11, Ame–μ15, Panda–22; Set 3: gpz–47, GPL–29, gpz–20; Set 4: GPL–47, GPL–60, gpz–6 [2,3]. PCR products meeting quality were genotyped under light–protected conditions. To enhance genotyping reliability, a multi–tube PCR approach was applied. According to the results of sex determination, bite section records from feces, and microsatellite profiles, individual identification was conducted by comparing against the existing giant panda DNA database from the Sichuan Liziping National Nature Reserve. GeneMarker V2.2.0 was used for genotyping of the initial detection results. After confirming genotypes, the Mstool plugin in Excel was employed for genetic comparison of the results. Two samples were considered to originate from the same individual if they matched at ≥10 of the 12 loci. Samples differing at only two loci (excluding cases involving special alleles) were also assigned to the same individual.

**Hormone assay**

Approximately 0.25–0.30g of feces was placed in a centrifuge tube, and methanol was added to extract hormones from the sample. The mixture was then incubated on a rotary shaker for 12–24 h to ensure complete hormone dissolution, followed by centrifugation at 3000 rpm for 10 min. The supernatant was then collected and assayed for estrogen, progesterone, cortisol, and testosterone concentrations using enzyme–linked immunosorbent assay (ELISA) kits according to the manufacturer’s instructions. All hormone concentrations were uniformly converted to ng/g dry fecal weight. Some testosterone samples that fell below the detection limit were assigned the minimum detection limit value, and these samples can be detected using other hormones in this study. For hormone-related analyses, only samples with complete measurements for all target hormones were retained; any samples with missing data were excluded. Following unit standardization, Z-score standardization was applied to the retained data.

**Data analysis**

Raw sequencing reads were processed using SMRT Link software (v8.0) with the following criteria: minPasses ≥5 and minPredictedAccuracy ≥0.9 to generate circular consensus sequencing (CCS) reads. Subsequently, Lima (v1.7.0) was used to demultiplex CCS reads into samples based on their barcode sequences. Reads lacking primer sequences or falling outside the expected length ranges (16S rRNA: 1,200–1,650 bp) were removed using Cutadapt. Chimera sequences were detected and eliminated using the UCHIME algorithm to yield final high–quality clean reads.

Microbiome bioinformatics analyses were conducted in QIIME 2 (version 2022.2) [4] with slight modification based on the official tutorials (https://docs.qiime2.org/2022.2/tutorials/). Briefly, raw sequencing reads were demultiplexed and then subjected to quality filtering, denoising, read merging, and chimera removal using the DADA2 plugin [5]. Resulting non–singleton amplicon sequence variants (ASVs) were aligned with MAFFT [6]. Taxonomy was assigned to ASVs using the classifysklearn naïve Bayes taxonomy classifier in the feature–classifier plugin [7] against the silva138 reference sequences [8]. The taxonomic composition from phylum to species level was visualized using Krona charts. Alpha diversity (Shannon index) and beta diversity (weighted UniFrac distance) were calculated using the diversity plugin. Weighted UniFrac distances generated in QIIME 2 were used to perform principal coordinate analysis (PCoA). Differential abundance of taxa across groups was tested with the Kruskal–Wallis test, and differentially abundant taxa were identified via linear discriminant analysis effect size (LEfSe), with an LDA score cutoff ≥ 2. Spearman’s rank correlation was applied to evaluate associations between hormone levels and gut microbial abundance (genus level) or alpha diversity. Significance thresholds were set at absolute correlation coefficient ≥ 0.25 with a p‑value ≤ 0.05. Significant associations were visualized as interaction networks in Cytoscape (v3.9.1), where nodes represent microbial taxa or hormones and edges denote significant correlations (red: positive, blue: negative). Hormones across months was visualized using the ggplot2 package in R. We compared the overall intra–group dissimilarity in hormone profiles (Euclidean distances) and microbiome composition (Bray–Curtis distances) in females and males, while regression analysis was performed to assess the relationship between hormone profile and gut microbiome.

**REFERENCES：**

1. Xu, Xiao, Yuzhi Li, Xiaofang Wang, Kun Wei, Wenping Zhang, Zhihe Zhang, Fujuan Shen, and Bisong Yue. 2010. “Zinc–finger intron 7: a new locus for sex identification of giant panda (*Ailuropoda melanoleuca*).” *Zoo Biology* 29: 526–531. https://doi.org/10.1002/zoo.20274

2. Lu, Zhi, Warren E. Johnson, Marilyn Menotti–Raymond, Naoya Yuhki, Janice S. Martenson, Susan Mainka, Huang Shi–Qiang, et al. 2001. “Patterns of genetic diversity in remaining giant panda populations.” *Conservation Biology* 15: 1596–1607. https://doi.org/10.1046/j.1523–1739.2001.00086.x

3. Huang, Jie, Yu–Zhi Li, Lian–Ming Du, Bo Yang, Fu–Jun Shen, He–Min Zhang, Zhi–He Zhang, Xiu–Yue Zhang, and Bisong Yue. 2015. “Genome–wide survey and analysis of microsatellites in giant panda (*Ailuropoda melanoleuca*), with a focus on the applications of a novel microsatellite marker system.” *BMC Genomics* 16: 61. https://doi.org/10.1186/s12864–015–1268–z

4. Philippot, Laurent, Aymé Spor, Catherine Hénault, David Bru, Florian Bizouard, Christopher M. Jones, Amadou Sarr, and Pierre–Alain Maron. 2013. “Loss in microbial diversity affects nitrogen cycling in soil.” *The International Society for Microbial Ecology Journal* 7: 1609–1619. https://doi.org/10.1038/ismej.2013.34

5. Rognes, Torbjørn, Tomáš Flouri, Ben Nichols, Christopher Quince, and Frédéric Mahé. 2016. “VSEARCH: a versatile open source tool for metagenomics.” *PeerJ* 4: e2584. https://doi.org/10.7717/peerj.2584

6. Katoh, Kazutaka, and Daron M. Standley. 2013. “MAFFT multiple sequence alignment software version 7: improvements in performance and usability.” *Molecular Biology and Evolution* 30: 772–780. https://doi.org/10.1093/molbev/mst010

7. Kaehler, Benjamin D., Nicholas A. Bokulich, Daniel McDonald, Rob Knight, J. Gregory Caporaso, and Gavin A. Huttley. 2019. “Species abundance information improves sequence taxonomy classification accuracy.” *Nature Communications* 10: 4643. https://doi.org/10.1038/s41467–019–12669–6

8. Glöckner, Frank Oliver, Pelin Yilmaz, Christian Quast, Jan Gerken, Alan Beccati, Andreea Ciuprina, Gerrit Bruns, et al. 2017. “25 years of serving the community with ribosomal RNA gene reference databases and tools.” *Journal of Biotechnology* 261: 169–176. https://doi.org/10.1016/j.jbiotec.2017.06.1198

**FIGURE S1 Variation in gut microbiome alpha diversity across months and between sexes in giant pandas.** ***p* < 0 .01. (A) Analysis of alpha diversity (Shannon index) in the gut microbiome of giant pandas across different months. (B–D) Comparison of alpha diversity (Shannon index) between female and male giant pandas in March (B), April (C), and November (D).

**FIGURE S2 Variation in gut microbiome beta diversity across months and between sexes in giant pandas.** **p* < 0.05, ****p* < 0.001, *****p* < 0.0001. (A) Principal coordinate analysis (PCoA) based on weighted UniFrac distances, showing gut microbial variation across months. (B) Intergroup differential analysis based on weighted UniFrac distances. (C and D) PCoA based on weighted UniFrac distances, illustrating sex–based differences in gut microbiome composition in March (C) and November (D).

**FIGURE S3 Variation in gut microbiome composition across months and between sexes in giant pandas.** (A) Gut microbiome composition at the genus level across different months in giant pandas. (B and C) Gut microbiome composition at the genus level in male (B) and female (C) giant pandas across different months. (D) Taxa with significant differences in the gut microbiome at the genus level. (E) Linear discriminant analysis Effect Size (LEfSe) was used to determine the significant difference in the abundance of gut microbiome between female and male giant pandas. (F and G) LEfSe was used to determine the significant difference in the abundance of gut microbiome between female and male giant pandas in March (F) and November (G).

**FIGURE S4. Hormone level variations in female and male giant pandas across different physiological stages.** EST, estrogen; PRO, progesterone; COR, cortisol; TOS, testosterone. **p* < 0.05, ***p* < 0 .01, ****p* < 0.001, ****p < 0.0001. (A–D) Monthly changes in estrogen (A), progesterone (B), cortisol (C) and testosterone (D) levels in female giant pandas. (E–H) Monthly changes in estrogen (E), progesterone (F), cortisol (G) and testosterone (H) levels in female giant pandas.

**FIGURE S5. Correlation network visualized by Cytoscape, illustrating interactions between gut microbial taxa and hormone levels in male giant pandas.**
